# Quantitative meniscus imaging and analysis: A narrative review

**DOI:** 10.1016/j.ostima.2025.100358

**Published:** 2025-06-18

**Authors:** Kalpana Sharma

**Affiliations:** a Research Program for Musculoskeletal Imaging, Center for Anatomy and Cell Biology, Paracelsus Medical University, Salzburg, Austria; b Department of Anatomy, Kathmandu University School of Medical Sciences, Dhulikhel, Nepal

**Keywords:** Meniscus, Magnetic resonance imaging, Quantitative analysis, Knee osteoarthritis, Progression, Knee pain

## Abstract

**Objective:**

In this review, we summarize the literature on the evolution of magnetic resonance imaging (MRI), segmentation, and quantitative analysis (qMRI) of the meniscus, while bearing in mind the pivotal role of the meniscus in the development (incidence) and progression of symptomatic and structural knee osteoarthritis (KOA).

**Design:**

We performed a literature search across PubMed and Google Scholar, spanning 35 years (1989–2024). We utilized keywords such as “meniscus”, “fibrocartilage”, “imaging”, “magnetic resonance”, “radiography”, “morphometry”, “quantitative analysis”, “knee”, “osteoarthritis”, “symptoms”, “pain”, “structure”, “progression”, “radiographic”, and “reproducibility”

**Results:**

Technological advances in image acquisition, segmentation, and derivation of quantitative analytic endpoints pertinent to meniscus morphometry (e.g., height, width, and volume) and position (e.g., tibial coverage, extrusion area, and distance) within the joint are highlighted in the literature. Three-dimensional qMRI of the meniscus has emerged as a reliable and reproducible non-invasive measurement technology, offering enhanced efficacy for assessing the relationship of the meniscus with radiographic joint space width (JSW), knee pain, structural (radiographic) KOA status, and with symptomatic and structural progression of KOA. Quantitative measures of meniscal extrusion were found to be robust predictors of various imaging endpoints, including osteophyte formation, subchondral bone changes, cartilage loss, as well as significant clinical outcomes.

**Conclusions:**

The emergence of quantitative meniscus measurement technology has revamped the field of meniscal imaging research, providing accurate 3D analysis of both morphometric and positional measures. The systematic application of this methodology has unveiled significant insights into a better understanding of the incidence and symptomatic and structural progression of KOA.

## 1 Introduction

Knee osteoarthritis (KOA) has been recognized as a debilitating, multifaceted whole-joint disease characterized by structural alterations in various tissues, including articular cartilage, menisci, subchondral and subarticular bone, capsule, synovial membranes, ligaments, adipose tissue, musculature, and others. As of 2020, an estimated 654 million adults aged 40 and above worldwide grapple with KOA. Globally, the prevalence of KOA is thought to be about 16 %, and the incidence rate is 203 per 10,000 person-years [1]. Within the spectrum of global disease burden, osteoarthritis (OA) ranked 17th in terms of prevalence amongst 369 disease entities in 2019 [2].

**Fig. 1:**
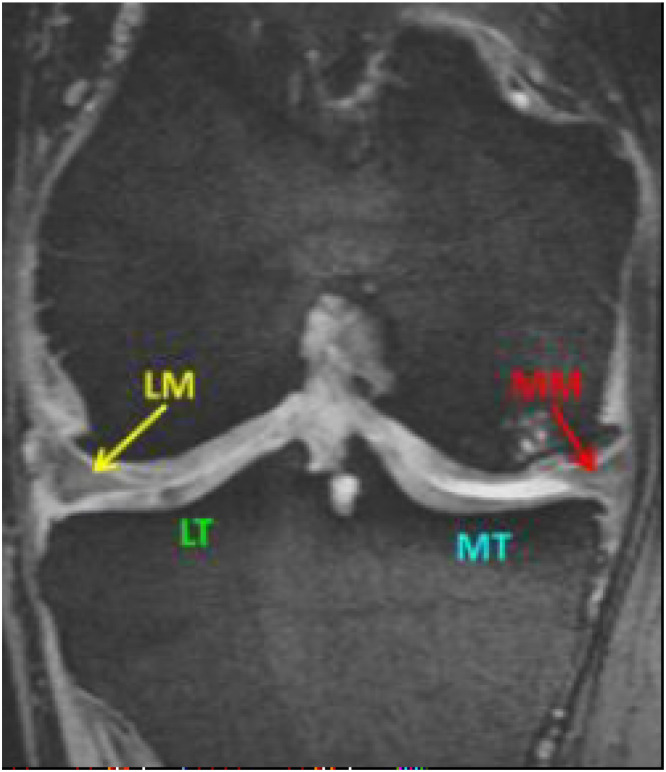
MRI showing the medial meniscus (MM), lateral meniscus (LM) and the cartilage surface areas as medial tibia (MT) and the lateral tibia (LT).

The medial and lateral menisci, located between the femoral condyles and the tibial plateau (Fig. 1), assume a crucial role in load distribution, shock absorption, stability, lubrication, proprioception, and overall knee joint function [3]. These crescent-shaped fibro cartilaginous structures play a key role in at least partially compensating for the incongruity of the rather flat articular surface of the proximal tibia and the relatively strong curvatures of femoral condyles in the coronal and particularly sagittal planes. Each meniscus consists of a central body and an anterior and posterior horn, each attached to the tibial plateau, connecting with the anterior cruciate ligaments (ACL) and posterior cruciate ligaments (PCL). The anterior horns of the medial and lateral menisci are linked by the so called transverse intermeniscal ligament [4]. The development of the meniscus begins in the 7th week of gestation with cell condensation, while blood vessels appear at the periphery of the meniscus by week 10 [5]. The developing menisci are highly cellular and vascularized during early gestation, with these features gradually diminishing at the later gestational and adult stages [6]. In adult life, the blood supply to the outer third of the meniscus remains at 10 to 33 %, which has important implications for healing [7]. The meniscus, when examined histologically, consists of three distinct layers. The superficial layer features a network of thin fibrils covering both the tibial and femoral surfaces. Beneath this lies a layer of lamella-like collagen fibrils that are radially oriented at the periphery of these surfaces. The central portion of the meniscus is composed of circularly arranged collagen fibrils between the femoral and tibial surface layers. This structural organization provides a functional explanation for the longitudinal orientation of most tears in meniscal tissue [8].

**Fig. 2:**
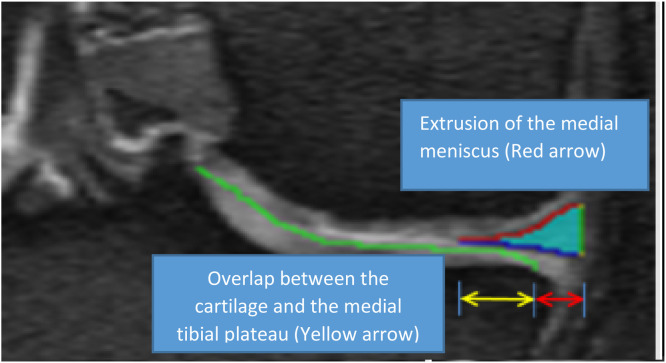
Coronal reconstruction of DESS MRI slice showing the extrusion of the medial meniscus (red arrow), indicating outward displacement of the meniscus beyond the tibial margin.

Meniscal damage, specifically tears and extrusion, is common in middle-aged and elderly populations [9], even in the absence of radiographic KOA. Medial meniscus tears, especially posterior root tears, are often degenerative and commonly found in middle-aged women, accounting for up to 21.5 % of posterior horn medial meniscus tears [10]. Meniscal extrusion, the outward displacement of the meniscus beyond the tibial margin (Fig. 2), is considered a significant risk factor for KOA [11]. Radial displacement of the meniscus results in the loss of meniscal function [12], contributing to knee pain [13] and eventually requiring knee replacement [14].

For many decades, radiography has been the gold standard for clinically diagnosing and monitoring the progression of KOA. This cost-effective and widely available technique is frequently employed to assess JSW, serving as an indirect indicator of cartilage and meniscus integrity. Several positioning protocols with fluoroscopy (semi-flexed, Schuss methods) [15,16] and without fluoroscopy (meta-tarso-phalangeal (MTP), fixed flexion methods) [17,18] have been developed in radiography to assess JSW. It is essential, however, to acknowledge its limitations in sensitivity and specificity, particularly in the longitudinal evaluation of KOA [19].

Various semi-quantitative scoring systems such as the Whole Organ Magnetic Resonance Imaging Score (WORMS) [20], Boston Leeds Osteoarthritis Knee Score (BLOKS) [21], and Magnetic Resonance Imaging Osteoarthritis Knee Score (MOAKS) [22] have been developed to evaluate knee joint pathology. These systems offer valid, reliable, and responsive tools for understanding the natural history of KOA and the structural aspects of disease onset and progression [23]. However, these reading systems deliver “scores of structural pathology” and do not permit 3D analysis of the meniscus; they, therefore, exhibit limited spatial accuracy in image assessment and limited sensitivity to longitudinal change. Thus, the advancement of 3D quantitative measurement technologies has significantly improved the ability to acquire 3D morphological and positional data for the menisci [24]. These technologies are highly sensitive for tracking longitudinal changes in KOA [25] and have a demonstrated relationship among biochemical, clinical, demographic, and imaging biomarkers for assessing disease severity and progression of KOA [26].

Since the beginning of this century, large public databases have been established to support osteoarthritis research. These include the Osteoarthritis Initiative (OAI), Multicenter Osteoarthritis Study (MOST), Osteoarthritis Data Integration Portal (OsteoDIP), the third National Health and Nutritional Examination Survey (NHANES-III), Health Improvement Network (THIN) and OAomics and molecular biomarkers (OAOB). Additionally, MR image repositories of KOA have been instituted that make clinical patient data, radiographic and MR images, and image measurements publicly available to interested researchers [27]. The most prominent among these is the OAI, a study on 4796 participants with, or at risk of KOA. Participants were initially followed over a 4-year period [28], with annual MRI assessments among many other measures, and then continued to be followed for up to 9 years [29]. Much of the work referenced here has emerged from this particular study. Furthermore, several initiatives have emerged to systematically analyze specific patients and patient groups from the OAI sample, including the Foundation of the National Institute of Health (NIH) Osteoarthritis Biomarker Consortium [30]. In this sample of 600 OAI participants, the relationships among a plethora of quantitative and semi-quantitative imaging biomarkers have been explored in terms of predicting or displaying association with symptomatic, structural (radiographic), or combined progression of KOA in relation to non-progressor knees [31].

In the current narrative review, we will synthesize the literature on the evolution of MR imaging, segmentation and quantitative analysis (qMRI) of the meniscus, cognizant of its pivotal role in the development (incidence) and progression of symptomatic and structural KOA.

## 2 Methods

The literature search was conducted using Pub Med and Google Scholar, spanning a 35-year period (1989–2024). The search terms used to identify relevant studies were “meniscus”, “fibrocartilage”, “imaging”, “magnetic resonance”, “radiography”, “morphometry”, “quantitative analysis”, “knee”, “osteoarthritis”, “symptoms”, “pain”, “structure”, “progression”, “radiographic”, and “reproducibility”. As this review primarily highlights methodological advancements in quantitative measures of meniscus morphology and position, it contains a high proportion of literature from our own research group. It does not include a greatly diverse range of perspectives and methodologies, and it does not represent a comprehensive summary of the entire literature on the meniscus. Approximately 300 papers were initially identified as suitable for the narrative review. These 300 were winnowed to 87 based on their relevance to the research topic, with a focus on literature related to 3D quantitative measurement of the meniscus, technological advancements in imaging, and the relationship of meniscus measures with joint space narrowing (JSN), knee pain, structural KOA status, as well as radiographic, symptomatic and, combined progression of KOA.

## 3 Results

### 3.1 Technological advances in quantitative imaging of the meniscus

#### 3.1.1 Meniscus image acquisition

Different MRI pulse sequences applied in different spatial orientations offer great sensitivity and specificity in diagnosing various knee joint pathologies, including those affecting the articular cartilage and meniscus [32,33]. An appropriate MRI pulse sequence must be chosen and tailored to each study [34]. Multiple imaging sequences, including routine spin-echo (SE) with different weightings, gradient-recalled acquisition in the steady state (GRASS), fast spin-echo (FSE), inversion recovery, 3D Fourier transform imaging (3DFT), and radial sequences, have all been utilized to examine meniscal pathologies [35]. Among these, FSE with proton-density weighted sequences have been favored for diagnosing meniscus tears [36]. Sensitivities for detecting medial meniscal tears have been reported to range from 86 to 96 % and specificities from 84 to 94 % [37].

The OAI MRI protocol has proposed the use of an intermediate-weighted 2D turbo spin echo (IW-TSE) sequence for visualizing knee joint structures and pathology, which is particularly adept at detecting meniscal tears [38]. The OAI MRI protocol further entails a 3D dual echo in steady state (DESS) sequence with water excitation (WE) with a low (0.7 mm) slice thickness and high (0.37 mm x 0.46 mm) in-plane resolution for superior cartilage visualization and for unveiling tears in the anterior and posterior horns of the menisci [38]. Further, due to its near-isotropic resolution, the DESS can be reformatted to the coronal plane (multi-planar reconstruction (MPR)), in which meniscal extrusion can be evaluated best [24]. Meniscal segmentation and morphometry derived from the DESS have exhibited strong agreement with measurements obtained from the coronal IW-TSE, demonstrating similar intra- and inter-observer reproducibility [39]. Although IW-TSE is well suited for visualizing meniscal lesions, DESS holds the benefit of superior spatial resolution and comes with the advantage of also depicting the tibial cartilage surface, bone interface, and cartilage thickness accurately [40,41], making it feasible not only to evaluate but also measure meniscal extrusion.

#### 3.1.2 Image segmentation

**Fig. 3:**
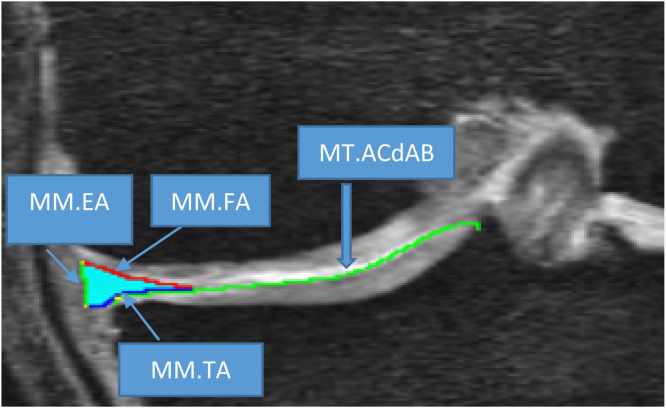
MRI showing manual segmentation of the medial meniscus, highlighting the meniscal areas (TA: tibial area; FA: femoral area; EA: external area) and cartilage surface area (ACdAB) of the MT.

The evolution of meniscal segmentation has transitioned from manual techniques to advanced automated methodologies. However, manual segmentation of the meniscus, delineating the meniscal areas such as the tibial area, femoral area, external area, and the cartilage surface area (ACdAB) of the tibia (Fig. 3), conducted by expert readers with secondary quality control checks, remains the gold standard in qMRI of the meniscus. Despite its accuracy, manual segmentation has drawbacks, including time-intensive processes, susceptibility to inter- and intra-observer variability, and the challenge of dealing with overlapping contrast behavior of surrounding tissues in MR images [42].

In the realm of meniscal segmentation, pioneering work was initiated by Sasaki et al. in 1999, employing a fuzzy rule-based approach. Although the segmentation success of the system was not quantitatively assessed, the analysis encompassed five datasets [43]. Subsequently, a semi-automated segmentation technique was devised to expedite the quantitative analysis of extensive datasets. While demonstrating acceptable accuracy and intra-reader variability, the method proved efficient in characterizing the meniscus in healthy and mild to moderate KOA cases, but was less than optimal for severe KOA or acute meniscal tears [42].

Numerous studies have investigated the development of automated techniques for segmenting the knee meniscus. Techniques such as extreme learning machines with discriminative random fields [44], deformable model approaches [45], multi-atlas rigid registrations [46], histogram of oriented gradients and local binary patterns [47], conditional generative adversarial networks [48], and convolutional neural networks (CNNs) [[49], [50], [51], [52]] have been employed across various investigations. These studies provide a foundation for future research in detecting meniscal tears and anomalies, offering effective diagnostic tools for orthopedic surgeons pre-arthroscopic surgery [51,52].

When comparing traditional machine learning models to deep learning techniques, traditional models rely on hand-crafted features and perform effectively with small datasets and simpler tasks. In contrast, deep learning models automatically learn features from raw data, making them highly effective for complex segmentation tasks. While deep learning demands large datasets and substantial computational power, it achieves results approaching expert-level accuracy and provides additional advantages such as 3D smoothness and scalability for analyzing larger cohorts [53].

#### 3.1.3 Quantitative imaging measures (morphometry and position)

The quantitative outcomes of meniscal measurements include the numerical assessment of various parameters such as meniscus position, morphology, and shape. A nomenclature for 3D parameters of meniscal position, morphology, and shape has been published earlier [24], which itself was based on a proposal for morphometric measures of cartilage [54] (Table 1).

**Table 1:** Nomenclature for describing 3D parameters of meniscal position, morphology and shape with measurement units.

Label	Explanations	Unit
ACdAB	Joint surface of the tibia, including the cartilage surface area (AC) and denuded areas of subchondral bone (dAB)	cm^2^
ACdAB.Cov	ACdAB covered by the meniscus	cm^2^
ACdAB.Cov %	Percentage of ACdAB covered by MM,TA	%
ACdAB.Uncov	ACdAB not covered by the meniscus	cm^2^
ACdAB.Uncov %	Percentage of ACdAB not covered by MM,TA	%
TA	Inferiorly located surface of the meniscus (oriented toward the tibia)	cm^2^
FA	Superiorly located surface of the meniscus (oriented toward the femur)	cm^2^
EA	External area of the meniscus (connection to the medial collateral ligament)	cm^2^
Th.Me	Mean thickness of the meniscus	mm
Th.Max	Maximum thickness of the meniscus	mm
V	Volume of the meniscus	mm^3^
Bul.Me	Mean bulging of the meniscus over all slices (2D measurement)	mm
Wid.Me	Mean width of the meniscus	mm
Wid.Max	Maximum width of the meniscus	mm
TA.Cov	MM,TA covered by the tibia	cm^2^
TA.Cov %	Percentage of MM,TA covered by the tibia	%
TA.Uncov	MM,TA not covered by the tibia	cm^2^
TA.Uncov %	Percentage of MM,TA not covered by the tibia	%
mEx.Me	Mean medial extrusion of the meniscus	mm
mEx.Min	Minimum medial extrusion of the meniscus	mm
mEx.Max	Maximum medial extrusion of the meniscus	mm
OvD.Me	Mean overlap distance ¼ distance between internal border of MM and external border of MT.ACdAB	mm
OvD.Min	Minimal overlap distance	mm
OvD.Max	Maximum overlap distance	mm

##### 3.1.3.1 3D quantitative measures of the meniscus

**Fig. 4:**
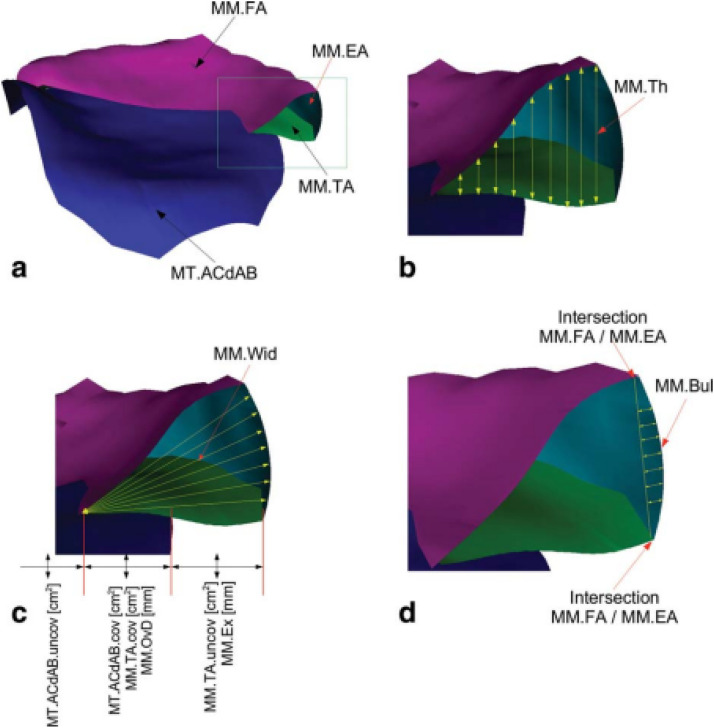
3D illustration of the medial meniscus: (a) depicting the medial tibial denuded areas of subchondral bone (MT.ACdAB, blue), medial meniscus femoral area (MM.FA, magenta), medial meniscus tibial area (MM.TA, green), and medial meniscus external area (MM.EA, turquoise); (b) illustrating the bidirectional measurement of medial meniscus thickness (MM.Th, yellow arrows); (c) showing the measurement of meniscal width (MM.Wid, yellow arrows). The overlap between MM.TA and MT.ACdAB was used to calculate surface coverage (MM.TA.Cov and MT.ACdAB.Cov in cm²), overlap distance (MM.OvD in mm), and extrusion (MM.Ex in mm); (d) depicting the measurement of medial meniscus bulging (MM.Bul, yellow arrows). Reproduced from Wirth et al. (2010) [24], with permission from John Wiley & Sons.

The relative position of the meniscus on the tibial plateau was indirectly identified by examining the overlap between the area of the cartilage surface, including denuded areas of subchondral bone (MT.ACdAB), and the medial meniscus tibial area (Fig. 4a). Meniscal extrusion was computed in the medial direction by extracting the external border of MT.ACdAB (Fig. 4a). The tibial plateau area was considered as the area of the cartilage surface, including denuded areas of subchondral bone (ACdAB) (Fig. 4a). Coverage of the tibial plateau (ACdAB.Cov) by the meniscus was calculated in absolute (mm^2^) and relative units (%) (Fig. 4c). The meniscal morphology was determined by computing the meniscal surfaces, volume, width (mean=Wid.Me and maximum=Wid.Max), thickness (mean=Th.Me and maximum=Th.Max) of the menisci (Fig. 4b-c). The borders of the menisci were identified by the internal margin of the cartilage surface of the medial tibia and lateral tibia. The volume of the medial meniscus was calculated by integrating the voxels within the femoral area, tibial area, and external area. The mean bulging of the external area was assessed slice by slice, measuring the distance between external area and a straight line connecting the intersection of the tibial and external areas with the intersection of the femoral and external areas [24] (Fig. 4d).

##### 3.1.3.2 Correlation of meniscal measures with a whole meniscus and central-5-slices

Manual segmentation for the quantitative measurement of the meniscus involved segmenting the cartilage surface (ACdAB) and meniscal areas (femoral, tibial and external, which commenced anteriorly and ended posteriorly, with approximately 45 and 90 min required for the medial and lateral meniscus, respectively. Measurements obtained from the central five coronal slices of the medial meniscus exhibited a high correlation with those obtained from the entire meniscus. This finding permits a reduction in analysis time for larger cohorts by focusing the segmentation on the central 5 slices [55].

##### 3.1.3.3 *Correlation between quantitative* vs *semi-quantitative measures*

Meniscal damage, particularly extrusion, has been assessed on MRI utilizing semi-quantitative scoring systems. Recognizing the inherent subjectivity in objectively evaluating meniscal extrusion and morphology via semi-quantitative scoring methods, recent advancements in quantitative technology have facilitated 3D measurement of meniscus position and morphology [24]. The findings reveal a moderate correlation between quantitative extrusion measures and semi-quantitative extrusion scores, indicating the methodological validity of quantitative extrusion measurements. Quantitative extrusion measures exhibit a stronger correlation with the tibial plateau coverage by the meniscus compared to semi-quantitative extrusion scores [56].

##### 3.1.3.4 Intra- and inter-observer reproducibility of meniscus measurement

Quantitative measurements of meniscus position and morphology were compared between intra-healthy and intra-OA knees. In intra-healthy knees, meniscal and tibial surface areas were found to be greater in men than in women. However, it was observed that "physiological" meniscal extrusion was greater in women than men [57]. Conversely, in intra-OA knees, those experiencing pain had less coverage of the medial tibial plateau and demonstrated greater medial meniscal extrusion compared to those without pain [58]. The developed 3D quantitative measure of meniscal position and morphology demonstrated good intra-operator test-retest reliability and satisfactory inter- and intra-observer reproducibility [39]. Furthermore, Katano et al. assessed interscan measurement errors by conducting two MRI examinations on the same day. The study reported that the relative interscan errors for meniscus measurements – including volume, area, coverage ratio, width, and thickness were <3 % for the entire medial and lateral meniscus [59].

### 3.2 Morphometric measurement of healthy menisci

#### 3.2.1 *Morphometric differences between the menisci (medial* vs *lateral)*

The medial meniscus displays a semilunar shape, characterized by a notably broader posterior end compared to its anterior counterpart. Conversely, the lateral meniscus assumes a more circular shape, with both ends exhibiting similar features [60]. Developmentally, observations during the initial 14 weeks of gestation indicate similar areas for both the medial and lateral femoro-tibial compartments. However, after this period, there was a discernible increase in the development of the medial tibial plateau in contrast to the lateral compartment. Consequently, it has been observed that the medial meniscus covers a smaller proportion of the medial tibial plateau than the lateral meniscus [61].

A detailed morphometric analysis of the medial and lateral meniscus revealed that the anterior and posterior thirds of the medial meniscus are thicker than their lateral counterparts. Notably, the posterior third represents the widest segment of the medial meniscus [62]. The distance between the anterior and posterior horns of the medial meniscus was found to be significantly greater than the lateral meniscus [63,64]. Both the outer and inner circumferences of the medial meniscus were notably greater than the lateral meniscus [64]. These observations collectively indicate that the medial meniscus covers a lesser proportion of the medial tibial plateau and exhibits a greater amount of physiological extrusion compared to the lateral meniscus [65].

#### 3.2.2 Correlation of meniscus measures with anthropometric variables (sex, age, weight, height, BMI)

Meniscus measurements have been found to correlate with various anthropometric factors such as age, sex, weight, height, and BMI. Notably, men exhibited significantly greater tibial meniscal surface areas, volume, and thickness. Interestingly, the same compartmental tibial plateau area demonstrated a stronger correlation with meniscal size in both genders, as opposed to body height or weight, with no significant correlation observed with age. Additionally, the tibial coverage by the meniscus was comparable between men and women [57]. However, it was observed that medial meniscal extrusion was higher in women compared to men, potentially attributed to variances in cartilage composition and gait mechanics [66]. Understanding the correlation of meniscal morphometric parameters with height, weight, and sex is crucial for appropriate meniscus transplantation. Notably, height displayed a strong correlation with total tibial plateau width, which in turn exhibited a strong correlation with all measurements of both medial and lateral menisci. Among these, the width of the medial meniscus demonstrated the highest correlation. It was observed that groups with higher BMI demonstrated significantly greater meniscal dimensions compared to those with lower BMI. This indicates that a greater BMI may contribute to higher mechanical loading and potentially altered biomechanics, which may impact the meniscal dimensions, necessitating surgical intervention [67,68].

#### 3.2.3 Correlation of meniscus measures with radiographic joint space width in healthy knees

The measurement of minimal JSW is noted to reflect a combination of cartilage and meniscal measures, particularly in women [69]. Thus, the significant contribution of the meniscus' position to minimal JSW raises concerns about the validity of JSW as an indirect measure of hyaline cartilage. The mean medial meniscus thickness in the posterior horn exhibited the strongest correlation with minimal JSW, followed by the mean medial thickness for the entire meniscus. Approximately three-quarters of meniscus position and morphology measurements demonstrated a statistically significant correlation with minimal JSW. The correlations between minimal JSW and meniscal measures were observed to be greater in women than men [69].

### 3.3 Relationship of meniscus measures with KOA

#### 3.3.1 Role of meniscus in joint space narrowing (JSN)

The incidence of meniscal damage, such as tears, extrusion, and subluxation, increases with a greater degree of JSN [70,71]. JSN, as evaluated through radiography, is primarily attributed to the loss of articular cartilage in the advanced stage of KOA. Prolonged knee pain has been linked to an increased risk of higher JSN grades [70]. Additionally, knees with JSN tend to cover a smaller tibial plateau area, indicating reduced mechanical protection of the knee by the meniscus. This results in greater meniscal extrusion, contributing to the onset of KOA [72].

#### 3.3.2 Correlation of meniscus measures with pain

Pathological changes in the meniscus, such as extrusion, tears, and degeneration, disrupt the biomechanical function of the knee joint, often resulting in knee pain and discomfort. Specifically, meniscal extrusion has shown a strong correlation with severe knee pain [13]. Understanding the relationship between meniscus measurements and knee pain is crucial in the treatment of KOA. It is important to acknowledge, however, that pain perception is subjective. Thus, the association between meniscal measurements and knee pain may vary among individuals [58]. A study by Wenger et al. identified a connection between frequent knee pain and meniscal extrusion, noting greater extrusion of the medial meniscus in painful knees compared to painless knees. The study revealed that in painful knees a lesser proportion of the medial tibial plateau is covered by the medial meniscus than in painless knees [58].

#### 3.3.3 T2, T1rho and other compositional measures

Early diagnosis of KOA hinges on the examination of biochemical alterations within the articular cartilage and menisci, characterized by the loss of glycosaminoglycans and degradation of collagen fibers [73]. Assessing the quality and biochemical composition of the meniscus and articular cartilage is paramount for identifying biomarkers associated with early KOA progression [74]. Techniques for compositional assessment, including T2 mapping, T1rho, and delayed gadolinium-enhanced MR imaging (dGEMRIC), enhance the evaluation of proteoglycans and collagen contents within these tissues [75,76].

Among these techniques, T1rho relaxation time stands out as a non-invasive method particularly sensitive to changes in proteoglycan contents of articular cartilage and menisci in early KOA. T2 relaxation time exhibits high sensitivity to changes in water content and collagen fiber orientation [77]. Gadolinium-enhanced MR imaging is highly sensitive to articular cartilage changes at the early stages of OA onset [75]. Ultrashort echo time-enhanced T2* mapping shows promise in detecting collagen organization within the meniscus and supporting the detection of subclinical meniscal degeneration [78]. These compositional imaging techniques provide insights into intra-meniscal changes at an early stage of KOA, offering a deeper understanding of the disease process well before morphological changes occur.

### 3.4 Longitudinal qMRI of the meniscus

#### 3.4.1 Longitudinal evaluation of meniscus measures (sensitivity to change in KOA)

Longitudinal assessment of the meniscus enables the evaluation of its structure, function, and pathological changes over a specific timeframe. Quantitative 3D measurements of medial meniscus position and morphology have revealed significant changes in rapidly progressing KOA, demonstrating sensitivity to change akin to cartilage and meniscal measures over a 2-year period. The study reported a robust correlation between meniscal measures and variations in change in radiographic JSW [79]. Sensitivity to change in meniscal measures was notably higher when evaluating the entire meniscus. The study showcased a longitudinal reduction in tibial plateau coverage by both the medial and lateral menisci in knees with medial JSN. This reduction in tibial plateau coverage by the medial meniscus was attributed to increased extrusion and decreased width [80].

#### 3.4.2 Relationship of the meniscus with structural KOA progression

Meniscal damage and extrusion have emerged as significant predictors of the structural progression of KOA. Additionally, following injury, meniscectomy has been identified as an important risk factor for structural progression. Structural progression of KOA is defined as the loss of ≥ 0.7 mm minimum JSW in the medial femorotibial compartment between baseline and 24, 36, or 48 months of follow-up. Measures of meniscal position, such as maximum extrusion distance of the total medial meniscus and mean extrusion in central or central-5-slices measures, were found to be higher in knees with medial compartment structural progression. Notably, 3D maximum and central medial meniscal extrusion played a vital role in the subsequent structural progression of KOA [55].

#### 3.4.3 Relationship of the meniscus with symptomatic KOA progression

Symptomatic progression of KOA was characterized by a persistent increase in the Western Ontario and McMaster Universities Osteoarthritis Index (WOMAC) pain subscale (≥9 on a 0–100 scale, transformed from the original 0–20 Likert scale used in the OAI) based on minimum clinically important difference (MCID) [81]. Approximately 75 % of patients with primary symptomatic KOA were found to have meniscal damage. Meniscal damage, including tears and extrusion, exhibited a strong association with cartilage loss and symptomatic KOA progression [82]. However, a recent study revealed that knees with isolated symptomatic progression displayed significantly less extrusion compared to knees with no progression. Moreover, greater medial meniscal extrusion was linked to combined structural and symptomatic KOA progression rather than isolated symptomatic KOA progression [25].

#### 3.4.4 Relationship of the meniscus with combined KOA progression (structural and symptomatic)

Meniscal extrusion disrupts the normal mechanical function of the meniscus and correlates with both structural and symptomatic changes in KOA [83]. Investigations have delved into the link between meniscal extrusions and isolated structural, isolated symptomatic or combined OA progression. Greater medial meniscal extrusion was found to be associated with combined structural and symptomatic progression of KOA. Notably, these associations were primarily influenced by structural progression rather than symptomatic progression. Baseline measures of tibial plateau coverage or overlap distance did not predict combined OA progression. However, a decline in tibial plateau coverage or overlap distance over a two-year period was indeed linked to combined OA progression [25].

#### 3.4.5 Relationship of meniscus with clinical outcomes, total knee replacement

Meniscal damage, particularly tears and extrusion, has been linked to the severity of KOA and the risk of total knee replacement (TKR) [14]. TKR is acknowledged as a cost-effective procedure and is considered the ultimate treatment for end-stage KOA. Studies have indicated that semi-quantitative scoring systems such as BLOKS and WORMS serve as independent predictors for TKR [84]. In a quantitative 3D measurement study, the association between meniscus position and morphology with the clinical progression of early KOA to TKR was assessed. The study revealed that a longitudinal increase in meniscal extrusion and reductions in tibial plateau coverage are the strongest predictors for TKR in rapidly progressing KOA. The study highlighted the finding that not only cartilage loss but also longitudinal changes in meniscus measurements can predict TKR [85].

The key findings on meniscus measurements and their relationships with KOA are summarized in Table 2.

**Table 2:** Key findings on meniscus measurements and relationships with KOA.

Meniscus measurement	Key findings
Meniscus image acquisition	OAI MRI protocol - IW-TSE and 3D DESS provide high sensitivity and specificity for detecting meniscal tears [37,38].
Image segmentation	Manual – gold standard but time intensive & observer dependent Automated/semi-automated – emerging techniques with high accuracy and efficient (deep learning) [42,[44], [45], [46], [47], [48], [49], [50], [51], [52]].
Quantitative imaging measures (position & morphology)	Meniscus position and morphology was assessed with parameters such as extrusion area, volume, width, thickness, and tibial plateau coverage [24]. Segmentation of central-5-slices correlates with whole meniscus, reducing analysis time [55]. Quantitative extrusion measures correlate moderately with semi-quantitative scores [56].
Morphometric differences between the menisci	Medial meniscus – semilunar in shape, greater extrusion than lateral meniscus Lateral meniscus – circular shape with more uniform dimensions [60].
Anthropometric correlations	Meniscus measurements correlates with age, sex, weight, height and BMI [57,67]. Meniscal extrusion is greater in women, potentially due to gait mechanics [66]. Higher BMI associated with larger meniscal dimensions, increasing risk to injury [67].
Correlation with JSW in healthy knees	Mean medial meniscus thickness in posterior horn shows strongest correlation with minimal JSW [69]. Correlations between minimal JSW and meniscal measures were observed to be greater in women [69].
Role of meniscus in joint space narrowing (JSN)	Meniscal damage correlates with increased JSN grades [70,71]. Knees with JSN exhibit reduced tibial plateau coverage by the meniscus, leading to meniscal extrusion and further progression of KOA [72].
Correlation with pain	Meniscal extrusion strongly correlates with knee pain severity [13]. Painful knees exhibit greater medial Meniscal extrusion and reduced tibial plateau coverage compared to painless knees [58].
Compositional measures	Techniques such as T2 mapping, T1rho and dGEMRIC assess meniscus composition and detect changes in early KOA [[75], [76], [77]].
Longitudinal qMRI of the meniscus (sensitivity to change)	Quantitative 3D measures of meniscus show sensitivity to changes in meniscal position and morphology [79]. Sensitivity to change in meniscal measures was higher when evaluating the entire meniscus [80].
Structural KOA progression	Greater medial meniscal extrusion predicts structural knee OA progression [55].
Symptomatic KOA progression	Symptomatic KOA progression often involves meniscal damage but shows less extrusion than structural progression [25].
Combined KOA progression	Greater medial meniscal extrusion associated with combined structural and symptomatic progression of KOA, with structural progression being the primary influence [25].
Clinical outcomes (TKR)	Meniscal extrusion and damage are strong predictors for TKR [14]. Longitudinal changes in meniscus measures predict TKR [85].

### 3.5 Challenges and future prospects

The increasing prevalence of obesity and an aging demographic presents substantial hurdles in KOA. The absence of reliable and responsive biomarkers persists as a major impediment to advancing the development of disease-modifying OA drugs. Conventional radiography, the prevailing benchmark for disease diagnosis and severity assessment, exhibits limited sensitivity to change and only modest correlation with clinical outcomes. Consequently, the quest for novel biomarkers to expedite the discovery of safe and efficacious treatments for KOA remains a formidable challenge.

Modern MRI techniques such as T1rho, T2 mapping, and delayed gadolinium-enhanced MR imaging (dGEMRIC) can assess the physiological and biochemical status of menisci and cartilage at early stages of KOA. Ultrashort echo time-enhanced T2* (UTE-T2*) mapping has introduced a novel technique to detect meniscal degeneration and collagen disorganization in early stages [78]. Automated segmentation methods such as deep learning and statistical shape models may play a pivotal role in future research on KOA. Statistical shape segmentation models, in particular, stand out for their ability to evaluate the 3D geometry of the meniscus and have been instrumental in identifying the association between geometric shape of the meniscus and KOA incidence [86]. Recently, statistical shape-modeling techniques have been used to examine meniscal shape alterations linked to the onset of tibial coverage loss and its association with structural progression in later OA stages [87].

## 4 Conclusion

The emergence of quantitative meniscal measurement technology has revamped the field of meniscus imaging research, providing accurate 3D analysis of both morphometric and positional measures. Systematic application of this methodology has unveiled significant insights into a better understanding of the incidence and symptomatic and structural progression of KOA. Despite significant advancements in MRI-based meniscus measurement, the lack of standardized and universally accepted quantitative meniscal measures remains a major challenge. Early diagnosis of KOA often relies on detecting biochemical alterations in the articular cartilage and menisci, such as glycosaminoglycan loss and collagen fibers degradation. While techniques such as T2 mapping, T1rho, delayed gadolinium-enhanced MR imaging show promise in evaluating proteoglycans and collagen content, they are not widely used in routine clinical practice. Additionally, the role of meniscus vascularization and nerve endings, which significantly impact pain perception and healing needs further investigation to enhance diagnostic and therapeutic approaches.

## Funding

This research did not receive any specific grant from funding agencies in the public, commercial, or not-for-profit sectors.

## Declaration of competing interest

None
